# Assessment of Seasonality and Extremely Preterm Birth in Denmark

**DOI:** 10.1001/jamanetworkopen.2021.45800

**Published:** 2022-02-03

**Authors:** Anders Hviid, Anna Laksafoss, Paula Hedley, Ulrik Lausten-Thomsen, Henrik Hjalgrim, Michael Christiansen, Sjurdur Frodi Olsen

**Affiliations:** 1 Department of Epidemiology Research, Statens Serum Institut, Copenhagen, Denmark; 2 Pharmacovigilance Research Center, Department of Drug Design and Pharmacology, University of Copenhagen, Copenhagen, Denmark; 3 Department for Congenital Disorders, Statens Serum Institut, Copenhagen, Denmark; 4 Department of Neonatology, Copenhagen University Hospital Rigshospitalet, Copenhagen, Denmark; 5 The Danish Cancer Society, Copenhagen, Denmark; 6 Department of Haematology, Rigshospitalet, Copenhagen, Denmark.; 7 Institute of Biomedical Sciences, University of Copenhagen, Copenhagen, Denmark

## Abstract

**Importance:**

Preterm birth, particularly extremely preterm birth, has been associated with substantial morbidity and mortality. Research during SARS-CoV-2–related lockdowns revealed reductions in the more severe subtypes of preterm birth in some countries, suggesting the presence of preventable risk factors, such as infectious diseases or social behavior. Seasonality may provide a similar means of assessing natural changes in the daily life of pregnant individuals that were similar to those experienced during the COVID-19 lockdown period.

**Objective:**

To evaluate the association between seasonality and extremely preterm birth.

**Design, Setting, and Participants:**

This nationwide cohort study included 1 136 143 pregnancies in Denmark with onset between January 1, 1997, and December 31, 2016, in which the fetuses survived 21 completed weeks of gestation. Pregnancies were followed up until preterm birth, fetal death, or 37 completed weeks of gestation. Data were analyzed from September 2020 to September 2021.

**Exposures:**

Season during gestation (primary exposure) and season of pregnancy onset.

**Main Outcomes and Measures:**

The main outcome of extremely preterm birth was defined as a live birth occurring between 22 weeks, 0 days’ gestation and 27 weeks, 6 days’ gestation. Cox regression analyses were used to estimate hazard ratios (HRs) for season during gestation and season of pregnancy onset, with adjustment for socioeconomic and demographic factors.

**Results:**

Among 662 338 pregnant individuals, the median age at pregnancy onset was 30.0 years (IQR, 6.0 years). Of 1 136 143 pregnancies, 2009 extremely preterm births (cumulative incidence, 0.18%) were identified during follow-up. Season during gestation was associated with extremely preterm birth, with cumulative incidences of 0.17% (95% CI, 0.16%-0.19%) in spring, 0.18% (95% CI, 0.17%-0.20%) in summer, 0.20% (95% CI, 0.18%-0.21%) in autumn, and 0.16% (95% CI, 0.14%-0.17%) in winter. Compared with winter, the adjusted HRs (AHRs) for the risk of extremely preterm birth were 1.11 (95% CI, 0.97-1.26) for spring, 1.15 (95% CI, 1.02-1.31) for summer, and 1.25 (95% CI, 1.10-1.42) for autumn. The number of extremely preterm births associated with the increased risk in the spring, summer, and autumn was 56.1 (95% CI, 18.2-99.7), representing 2.8% (95% CI, 0.9%-5.0%) of all extremely preterm births in the study. Season of pregnancy onset was not associated with the risk of extremely preterm birth in spring (AHR, 0.98; 95% CI, 0.95-1.01) or summer (AHR, 1.00; 95% CI, 0.96-1.03) compared with winter, but a slight increase in risk was observed in autumn (AHR, 1.05; 95% CI, 1.02-1.09) compared with winter.

**Conclusions and Relevance:**

In this large, national cohort study, seasonality was associated with 2.8% of all extremely preterm births. Season during gestation was associated with the rate of extremely preterm birth, suggesting the presence of potential risk factors associated with season that may be preventable. Further research to identify risk factors for extremely preterm birth associated with seasonality is warranted.

## Key Points

**Question:**

Is seasonality a risk factor associated with extremely preterm birth?

**Findings:**

In this cohort study of more than 1 million pregnancies involving 662 338 pregnant individuals in Denmark, season during gestation was associated with extremely preterm birth. Gestation during autumn was associated with the highest rate of extremely preterm birth, whereas gestation during winter was associated with the lowest rate.

**Meaning:**

This study found that season during gestation was associated with extremely preterm birth, suggesting the presence of risk factors associated with seasonality that may be preventable.

## Teaser

This cohort study examines the association between seasonality and extremely preterm birth among pregnant individuals in Denmark.

## Introduction

Preterm birth is the primary cause of death among children younger than 5 years.^1^ The severe subtypes of preterm birth (ie, very preterm and extremely preterm birth) are particularly associated with adverse outcomes. In France, extremely preterm birth has been associated with moderate to severe neurodevelopmental disabilities in 28% of children at age 5 years.^2^ In a Swedish cohort of children born extremely preterm between 2014 and 2016, the 1-year survival rate without any substantial morbidities was only 38%,^3^ and the adverse health consequences appeared to persist into adulthood.^4^ The identification of preventable risk factors for preterm births, particularly extremely preterm births, is of major public health importance.

The etiologic characteristics of preterm birth are, however, complex.^5^ Genetic factors, maternal characteristics, previous pregnancy history, and fetal, psychosocial, environmental, and pregnancy characteristics all appear to be associated with the risk of preterm birth, but few modifiable risk factors have been identified.^6^ Notably, in a recent study conducted in Denmark,^7^ a marked decrease in the prevalence of extremely preterm births was observed during the COVID-19 lockdown period between March 12 and April 14, 2020, compared with the same 1-month period in 2015 to 2019. This decrease was not found among very premature or moderately premature births, and there was no corresponding increase in the rate of stillbirths.^8^ Reductions in preterm birth after national lockdowns have been observed in several other countries, including Argentina, Australia, China, Ireland, Italy, the Netherlands, and the US,^9,10,11,12,13,14,15,16^ but these reduction have not been reported in all countries,^17,18^ and notably have not occurred in Sweden, where the COVID-19 lockdown restrictions were less severe.^19^ A meta-analysis found an overall reduction in preterm births in high-income but not low-income countries.^20^

The Danish observation of a marked decrease in extremely preterm births only could suggest that the COVID-19 lockdown had implications for risk factors that are specific to this phenotype to a greater extent than other preterm birth phenotypes. The most likely consequences of the lockdown were increases in focus on hygiene, working from home, and social distancing, potentially producing reductions in microbial exposure, changes in physical activity patterns, decreases in exposure to psychological stressors, and possible reductions in climate exposure. These and other profound changes in the daily lives of pregnant individuals may have constituted a unique natural phenomenon that facilitated the observed reduction in extremely preterm births. Seasonality appears to provide similar, although less extreme, changes in physical activity, climate exposure, and microbial exposure; thus, we hypothesized that seasonality might be associated with the risk of extremely preterm birth.

To evaluate this hypothesis, we conducted a large nationwide cohort study of the seasonality of extremely preterm birth that comprised all pregnancies in Denmark between 1997, and 2016. The data permitted us to distinguish the association of season at pregnancy onset from the association of season during gestation, to discern different obstetric subtypes of preterm birth, and to identify associations with extremely preterm birth compared with very preterm and moderately preterm birth.

## Methods

Ethics approval was not required because the study used data from administrative registers and was therefore exempt from providing informed consent according to regulations for register-based studies in Denmark. This study followed the Strengthening the Reporting of Observational Studies in Epidemiology (STROBE) reporting guideline for cohort studies.

### Pregnancy Cohort

We constructed a nationwide cohort of all recorded singleton pregnancies in Denmark with onset between January 1, 1997, and December 31, 2016, in which the fetuses survived 21 completed weeks of gestation. Data were analyzed from September 2020 to September 2021. We identified pregnancies resulting in singleton live births or stillbirths through the Danish Medical Birth Register.^21^ Pregnancies with abortive outcomes (spontaneous abortions, induced abortions, and other abortive outcomes, such as molar pregnancy and abnormal products of gestation) were identified using diagnostic codes from the *International Classification of Diseases, Tenth Revision* recorded in the National Hospital Register^22^ (eTable 1 in the Supplement). Timing of pregnancy onset was calculated by subtracting the recorded gestational age (in days) at birth or abortion from the date of birth or abortive outcome. Records of gestational ages at birth are based mainly on ultrasonographic results,^23^ whereas records of gestational age for abortive outcomes are based on either ultrasonographic results or the first day of the last menstrual period.

### Outcomes

Any preterm birth was defined as a live birth occurring between 22 weeks, 0 days’ gestation and 36 weeks, 6 days’ gestation. Extremely preterm birth was defined as a live birth occurring between 22 weeks, 0 days’ gestation and 27 weeks, 6 days’ gestation; very preterm as a live birth occurring between 28 weeks, 0 days’ gestation and 31 weeks, 6 days’ gestation; and moderately preterm as a live birth occurring between 32 weeks, 0 days’ gestation and 36 weeks, 6 days’ gestation. The analyses of very preterm and moderately preterm births were conditional on the fetus surviving 27 and 31 completed weeks of gestation, respectively. For further clinical subtyping of preterm births, we obtained information on possible cesarian delivery, induced birth, and premature prelabor rupture of membranes.

### Covariates

From Statistics Denmark and the Central Person Register, we obtained information on a number of demographic and socioeconomic covariates, including maternal employment status (employed, employed in a management position, self-employed, or unemployed and receiving public assistance), family structure (married, single, or living with partner), level of education (primary, secondary, postsecondary, or vocational school), disposable household income (quartile 1, 2, 3, or 4), location of residence in Denmark (capital region, middle region, northern region, Sealand, or southern region), and maternal place of birth (Denmark, Europe, or other).^24^ Data on race and ethnicity were not available. For pregnancies resulting in live births or stillbirths, we obtained information on maternal prepregnancy body mass index (calculated as weight in kilograms divided by height in meters squared) and smoking during pregnancy from the Danish Medical Birth Register.^21^ We also included information on parity (defined as previous pregnancies with at least 21 completed weeks of gestation). Because missing values were rare for most covariates, we used complete case analysis. Missing values were present to a greater extent for body mass index and smoking status; therefore, these covariates were only included in a sensitivity analysis of a subcohort with complete information.

### Statistical Analysis

We analyzed the pregnancy cohort using survival analysis with a fetuses-at-risk approach. All pregnancies with fetuses surviving 21 completed weeks of gestation were followed up from week 22 to birth, to week 36 and 6 days, or to December 31, 2016, whichever event occurred first. In the analysis of preterm birth subtypes, follow-up was concluded before 28 completed weeks of gestation for extremely preterm births, before 32 completed weeks of gestation for very preterm births, and before 37 completed weeks of gestation for moderately preterm births. Stillbirths and abortive outcomes occurring during follow-up were considered competing risks. Follow-up time was classified according to season during gestation, season of pregnancy onset, and covariates of interest. Seasons were defined as winter (December, January, and February), spring (March, April, and May), summer (June, July, and August), and autumn (September, October, and November).

We used Cox proportional hazards regression analyses with gestational age as the underlying time scale to estimate hazard ratios (HRs) assessing the risk of preterm birth according to season and month during gestation as well as season and month of pregnancy onset. Potential confounding covariates were included directly in the adjusted regression models. Continuous covariates, such as birth year and maternal age, were modeled using restricted cubic splines. We used the Aalen-Johansen estimator, taking into account competing risks to estimate the cumulative incidences of study outcomes. We estimated the absolute impact of season as the unadjusted observable proportion of potentially associated preterm births.^25^ Bootstrapping was used to derive 95% CIs.

Our main analysis assessed the association between season during gestation and extremely preterm birth. Assessments of the month during gestation and the season and month of pregnancy onset were considered secondary analyses. Very preterm and moderately preterm birth were considered comparative outcomes. We conducted an exploratory interaction analysis of gestation month and pregnancy onset month, and we performed several sensitivity analyses of data from the main analysis.

Data were analyzed using R software (R Foundation for Statistical Computing). The threshold for statistical significance was 2-sided *P* = .05.

### Results

We identified 1 577 511 pregnancies with onset between 1997 and 2016. Of those, 402 945 pregnancies (149 241 spontaneous abortions, 243 319 induced abortions, and 10 385 other abortive outcomes, such as molar pregnancy and abnormal products of gestation) had fetuses who did not survive 21 completed weeks of gestation. Among the resulting 1 174 566 pregnancies, 38 423 (3.2%) were excluded due to missing values (1.6% had missing information on disposable household income, 2.7% had missing information on maternal educational level, 0.9% had missing information on family structure, and <0.01% had missing information on maternal employment status). Of the resulting 1 136 143 pregnancies involving 662 338 individuals (median age at pregnancy onset, 30.0 years [IQR, 6.0 years]), we identified 2009 extremely preterm births over 6 777 673 fetal weeks of follow-up, 4746 very preterm births over 4 504 907 fetal weeks of follow-up, and 31 384 moderately preterm births over 5 560 081 fetal weeks of follow-up. The preterm birth prevalence was 3.4% for any preterm birth, 2.8% for moderately preterm birth, 0.4% for very preterm birth, and 0.2% for extremely preterm birth.

Extremely preterm births had covariate characteristics that were broadly similar to those of very preterm and moderately preterm births (Table 1). Most mothers were younger than 18 years at pregnancy onset (extremely preterm: 520 mothers [25.9%]; very preterm: 1271 mothers [26.8%]; moderately preterm: 8377 mothers [26.7%]), employed (extremely preterm: 1124 mothers [55.9%]; very preterm: 2691 mothers [56.7%]; moderately preterm: 17 950 mothers [57.2%]), and living with a partner (extremely preterm: 794 mothers [39.5%]; very preterm: 2012 mothers [42.4%]; moderately preterm: 13 786 mothers [43.9%]).

**Table 1.** Characteristics of Preterm Births^a^

Characteristic	Extremely preterm		Very preterm		Moderately preterm	
	No. (%)	Fetal weeks of follow-up, millions	No. (%)	Fetal weeks of follow-up, millions	No. (%)	Fetal weeks of follow-up, millions
Total preterm births, No.	2009	6.77	4746	4.50	31 384	5.56
Maternal employment status						
Employed	1124 (55.9)	3.87	2691 (56.7)	2.57	17 950 (57.2)	3.17
Employed, management position	203 (10.1)	0.96	527 (11.1)	0.64	3452 (11.0)	0.79
Self-employed	34 (1.7)	0.14	102 (2.1)	0.10	660 (2.1)	0.12
Unemployed and receiving public assistance	648 (32.3)	1.80	1426 (30.0)	1.20	9322 (29.7)	1.48
Family structure						
Married	742 (36.9)	2.74	1664 (35.1)	1.82	10 912 (34.8)	2.25
Single	473 (23.5)	1.20	1070 (22.5)	0.80	6686 (21.3)	0.98
Living with partner	794 (39.5)	2.83	2012 (42.4)	1.88	13 786 (43.9)	2.32
Maternal educational level						
Primary school	583 (29.0)	1.36	1270 (26.8)	0.90	7942 (25.3)	1.11
Secondary school	212 (10.6)	0.85	500 (10.5)	0.56	3622 (11.5)	0.70
Postsecondary school	620 (30.9)	2.59	1551 (32.7)	1.72	10 057 (32.0)	2.13
Vocational school	594 (29.6)	1.98	1425 (30.0)	1.32	9763 (31.1)	1.62
Household disposable income quartile						
1	673 (33.5)	1.73	1447 (30.5)	1.15	9161 (29.2)	1.41
2	633 (31.5)	2.17	1567 (33.0)	1.44	10 587 (33.7)	1.78
3	471 (23.4)	1.80	1143 (24.1)	1.20	7570 (24.1)	1.48
4	232 (11.5)	1.08	589 (12.4)	0.72	4066 (13.0)	0.89
Maternal place of birth						
Denmark	1698 (84.5)	5.95	4165 (87.8)	3.95	27 731 (88.4)	4.88
Europe	118 (5.9)	0.36	234 (4.9)	0.24	1468 (4.7)	0.30
Other	193 (9.6)	0.47	347 (7.3)	0.31	2185 (7.0)	0.38
Year of pregnancy onset						
1997-2000	341 (17.0)	1.40	1047 (21.4)	0.93	6392 (20.4)	1.15
2001-2004	415 (20.7)	1.41	1064 (22.4)	0.93	6944 (22.1)	1.15
2005-2008	431 (21.5)	1.41	974 (20.5)	0.94	6598 (21.0)	1.16
2009-2012	401 (20.0)	1.30	892 (18.8)	0.87	5830 (18.6)	1.07
2013-2016	421 (21.0)	1.16	769 (16.2)	0.79	5620 (17.9)	1.00
Maternal age at pregnancy onset, y						
<18	520 (25.9)	1.58	1271 (26.8)	1.05	8377 (26.7)	1.29
18-24	400 (19.9)	1.55	968 (20.4)	1.03	7014 (22.3)	1.27
25-34	252 (12.5)	1.10	658 (13.9)	0.73	4617 (14.7)	0.90
35-44	363 (18.1)	1.32	880 (18.5)	0.88	5396 (17.2)	1.08
≥45	474 (23.6)	1.23	969 (20.4)	0.82	5980 (19.1)	1.01
BMI						
<18.5	63 (3.1)	0.18	158 (3.3)	0.12	1112 (3.5)	0.15
18.5-25.0	670 (33.3)	2.60	1480 (31.2)	1.73	11 187 (35.6)	2.14
>25.0	492 (24.5)	1.40	1010 (21.3)	0.93	6434 (20.5)	1.15
Unknown	784 (39.0)	2.59	2098 (44.2)	1.72	12 651 (40.3)	2.12
Smoking status						
Nonsmoker	1291 (64.3)	5.23	3103 (65.4)	3.48	22 220 (70.8)	4.30
Stopped smoking during pregnancy	53 (2.6)	0.16	101 (2.1)	0.11	789 (2.5)	0.13
Smoker	359 (17.9)	0.84	882 (18.6)	0.56	5359 (17.1)	0.69
Unknown	306 (15.2)	0.54	660 (13.9)	0.36	3016 (9.6)	0.44

Abbreviations: BMI, body mass index (calculated as weight in kilograms divided by height in meters squared); NA, not applicable.

^a^ Among 1 136 143 pregnancies in Denmark with onset between January 1, 1997, and December 31, 2016.

**Figure 1.**
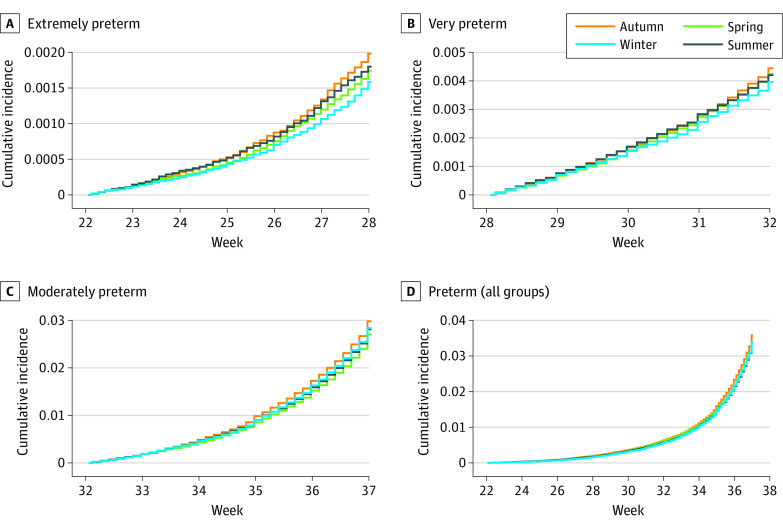
Cumulative Incidence of Preterm Birth According to Gestational Age at Birth and Season During Gestation Preterm births among 1 136 143 pregnancies in Denmark between January 1, 1997, and December 31, 2016, were included. Incidences were calculated among pregnancies with fetuses surviving 21, 27, and 31 completed weeks of gestation using the Aalen-Johansen estimator. For the combined preterm group, cumulative incidence was calculated for pregnancies with fetuses surviving 21 completed weeks of gestation. Extremely preterm was defined as a live birth occurring between 22 weeks, 0 days’ gestation and 27 weeks, 6 days’ gestation; very preterm as a live birth occurring between 28 weeks, 0 days’ gestation and 31 weeks, 6 days’ gestation; moderately preterm as a live birth occurring between 32 weeks, 0 days’ gestation and 36 weeks, 6 days’ gestation; and preterm (all groups) as a live birth occurring between 22 weeks, 0 days’ gestation and 36 weeks, 6 days’ gestation.

The cumulative incidence of extremely preterm birth was lowest in winter (0.16%; 95% CI, 0.14%-0.17%) and highest in autumn (0.20%; 95% CI, 0.18%-0.21%), followed by summer (0.18%; 95% CI, 0.17%-0.20%) and spring (0.17%; 95% CI, 0.16%-0.19%) (Figure 1). This pattern was attenuated as gestational age increased, and for the incidence of preterm birth overall, there was little evidence of seasonality (Figure 1). Comparing the season during gestation with the reference season of winter yielded adjusted HRs (AHRs) for the risk of extremely preterm birth of 1.11 (95% CI, 0.97-1.26) for spring, 1.15 (95% CI, 1.02-1.31) for summer, and 1.25 (95% CI, 1.10-1.42) for autumn (Table 2). A test for associations across seasons produced results that were not statistically significant. A similar but attenuated pattern was observed for the risk of very preterm birth (autumn vs winter: AHR, 1.13; 95% CI, 1.04-1.22). However, for the risk of moderately preterm birth, there was little evidence of seasonality (Table 2). The number of extremely preterm births associated with the increased risk in spring, summer, and autumn was 56.1 (95% CI, 18.2-99.7), representing 2.8% (95% CI, 0.9%-5.0%) of all extremely preterm births in the study. In the analysis of season of pregnancy onset, compared with winter, spring had the highest risk of extremely preterm birth (AHR, 1.12; 95% CI, 0.99-1.27), and summer had the lowest risk (AHR, 0.87; 95% CI, 0.77-0.99) (Table 2).

**Table 2.** Association of Season During Gestation and Season of Pregnancy Onset With Risk of Preterm Birth^a^

Season	Births, No. (fetal weeks of follow-up, millions)	Hazard ratio (95% CI)	
		Unadjusted	Adjusted^b^
**Gestation**			
Extremely preterm birth			
Winter	441 (1.7)	1 [Reference]	1 [Reference]
Spring	531 (1.9)	1.10 (0.98-1.25)	1.11 (0.97-1.26)
Summer	507 (1.7)	1.12 (0.99-1.27)	1.15 (1.02-1.31)
Autumn	530 (1.7)	1.22 (1.08-1.38)	1.25 (1.10-1.42)
Very preterm birth			
Winter	1093 (1.1)	1 [Reference]	1 [Reference]
Spring	1265 (1.2)	1.08 (1.00-1.17)	1.08 (0.99-1.17)
Summer	1220 (1.2)	1.07 (0.99-1.16)	1.06 (0.98-1.15)
Autumn	1168 (1.1)	1.13 (1.04-1.22)	1.13 (1.04-1.22)
Moderately preterm birth			
Winter	7537 (1.4)	1 [Reference]	1 [Reference]
Spring	7776 (1.5)	0.98 (0.95-1.01)	0.98 (0.95-1.01)
Summer	8354 (1.5)	1.01 (0.98-1.04)	1.00 (0.96-1.03)
Autumn	7717 (1.3)	1.05 (1.02-1.09)	1.05 (1.02-1.09)
**Pregnancy onset**			
Extremely preterm birth			
Winter	503 (1.7)	1 [Reference]	1 [Reference]
Spring	545 (1.6)	1.11 (0.99-1.26)	1.12 (0.99-1.27)
Summer	443 (1.7)	0.87 (0.76-0.99)	0.87 (0.77-0.99)
Autumn	518 (1.8)	0.96 (0.85-1.09)	0.96 (0.85-1.08)
Very preterm birth			
Winter	1192 (1.1)	1 [Reference]	1 [Reference]
Spring	1178 (1.1)	1.01 (0.93-1.10)	1.02 (0.94-1.10)
Summer	1141 (1.1)	0.94 (0.87-1.02)	0.94 (0.87-1.02)
Autumn	1235 (1.1)	0.96 (0.89-1.04)	0.96 (0.88-1.04)
Moderately preterm birth			
Winter	7803 (1.4)	1 [Reference]	1 [Reference]
Spring	7770 (1.3)	1.02 (0.99-1.05)	1.02 (0.99-1.05)
Summer	7622 (1.4)	0.95 (0.92-0.99)	0.96 (0.93-0.99)
Autumn	8180 (1.5)	0.97 (0.94-1.00)	0.97 (0.94-1.00)

^a^ Among 1 136 143 pregnancies in Denmark with onset between January 1, 1997, and December 31, 2016.

^b^ Adjusted for maternal employment status (employed, employed in a management position, self-employed, or unemployed and receiving public assistance), family structure (married, single, or living with partner), maternal educational level (primary, secondary, postsecondary, or vocational school), household income (quartile 1, 2, 3, or 4), maternal place of birth (Denmark, Europe, or other), location of maternal residence in Denmark at onset of pregnancy (capital region, middle region, northern region, sea land, or southern region) and the calendar year, and maternal age in years as restricted cubic splines.

**Figure 2.**
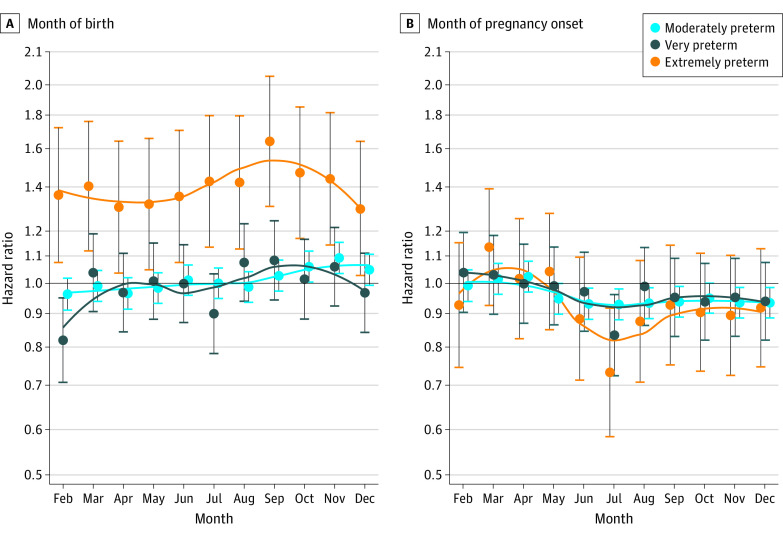
Preterm Birth According to Month During Gestation and Month of Pregnancy Onset Preterm births among 1 136 143 pregnancies in Denmark between January 1, 1997, and December 31, 2016, were included. January was used as the reference for both month of pregnancy onset and month during gestation. Dots represent hazard ratio point estimates, and whiskers represent 95% CIs.

**Figure 3.**
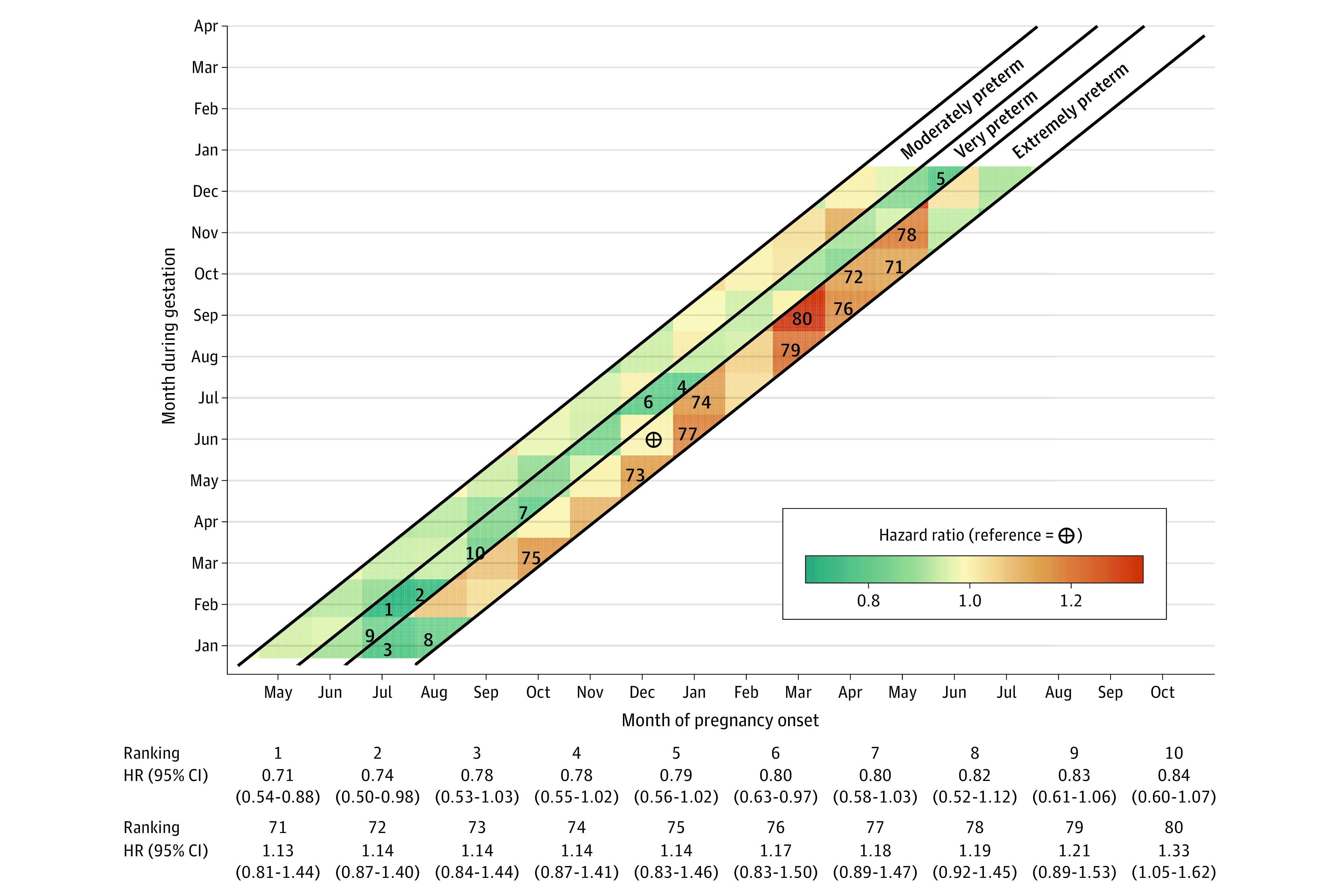
Heat Map of Preterm Birth According to Combinations of Month During Gestation and Month of Pregnancy Onset Preterm births among 1 136 143 pregnancies in Denmark between January 1, 1997, and December 31, 2016, were included. Gestation during June with pregnancy onset in December was used as the reference period. The 10 ratios with the highest increase (ranking 71-80) and the 10 with the highest decrease (ranking 1-10) compared to the reference period were numbered.

When comparing month during gestation with the reference month of January, we observed the highest risk of extremely preterm birth in September, October, and November; these monthly patterns were similarly attenuated for very preterm and moderately preterm births (Figure 2). Furthermore, among the winter months, January had a significantly lower risk of extremely preterm birth compared with December and February (Figure 2). In the analyses of month of pregnancy onset, the greatest risk of extremely preterm birth occurred in March, April, and May; these monthly patterns were also attenuated for very preterm and moderately preterm births (Figure 2). In an exploratory interaction analysis, pregnancies with onset in March, April, and May and gestation between August and November had the highest risk of extremely preterm birth (Figure 3).

In the cohort with complete information on body mass index and smoking status during pregnancy (n = 689 680), we assessed the association between season during gestation and risk of extremely preterm birth. This approach yielded AHRs similar to those observed in the main analysis (spring: AHR, 1.03 [95% CI, 0.87-1.22]; summer: AHR, 1.09 [95% CI, 0.92-1.29]; autumn: AHR, 1.22 [95% CI, 1.03-1.44]) (eTable 2 in the Supplement). Including only extremely preterm births recorded as spontaneous (n = 811) and considering cesarian delivery (n = 665), induced birth (n = 73), and premature prelabor rupture of membranes (n = 460) as competing risks yielded AHRs comparable with those found in the main analysis (spring: AHR, 1.20 [95% CI, 0.98-1.47]; summer: AHR, 1.30 [95% CI, 1.06-1.59]; autumn: AHR, 1.35 [95% CI, 1.11-1.66]) (eTable 2 in the Supplement). Adjusting for interpartum duration in the cohort with complete information on this variable (n = 1 123 956) or excluding pregnancies with short interpartum duration (n = 40 078) did not produce risk estimates that differed substantially from those in the main analysis (eTable 2 in the Supplement). Including possible diagnoses of preeclampsia as a time-varying covariate produced no change from the risk estimates observed in the main analysis (eTable 2 in the Supplement). However, excluding pregnancies with preeclampsia diagnoses (n = 32 543) attenuated the risk ratios (eg, AHR, 1.14; 95% CI, 1.04-1.25 for autumn vs winter) (eTable 2 in the Supplement).

## Discussion

In this cohort study involving more than 1 million pregnancies in Denmark, we observed an association between season during gestation and extremely preterm birth. Winter was associated with the lowest rate of extremely preterm birth, whereas autumn and summer were associated with the highest. A similar association between season during gestation and rate of very preterm birth was found. However, when all preterm subtypes were combined, the rate of preterm birth overall was not associated with season.

Several studies^26,27,28^ from low-, middle-, and high-income countries have reported seasonal patterns in the prevalence of preterm birth. In high-income countries, such as Japan and the US, winter and summer peaks in prevalence have been reported,^27,28^ whereas in a London-based cohort study, only a winter peak was observed.^26^ Studies included in a systematic review^29^ focusing on meteorological variables, such as temperature and humidity, have in many settings reported higher risks of preterm birth associated with increasing temperature and exposure to heat waves. In a comprehensive meta-analysis of European birth cohorts,^30^ the opposite pattern emerged; colder temperatures during the first trimester were associated with increases in the risk of preterm birth. However, this association was not found in the Danish National Birth Cohort, which was included in the meta-analysis.^30^ Between-country differences were not unexpected because of geographical, socioeconomic, and cultural differences. Denmark has a temperate climate, with few episodes of temperature extremes in either direction. Nevertheless, summer and autumn have higher daily mean temperatures than spring and winter. Our results were, to some extent, consistent with both higher temperatures during months of gestation and lower temperatures during months of pregnancy onset, which may be associated with increases in the risk of extremely preterm birth.

High levels of air pollution have been associated with preterm birth. For example, air pollution in the form of fine particulate matter and ozone exposure during pregnancy appears to be associated with consistent increases in the risk of preterm birth in many settings.^31^ Notably, a study differentiating between preterm birth subtypes found that areas with the highest levels of air pollutant exposure were associated with the greatest number of very preterm births.^32^ Compared with other European countries, the air quality in Denmark compares well, and air pollutants are therefore unlikely to explain the observed pattern of preterm birth in the country.^33^

Seasonality has also been associated with a number of changes in individual behavior and societal practices, many of which mimic the changes imposed by the SARS-CoV-2–related national lockdowns, although to a lesser extent. Moderate levels of physical activity, particularly in the form of leisure activity before and during pregnancy, appear to be protective against preterm birth compared with inactivity and higher levels of physical activity.^34^ Few studies have distinguished between clinical subtypes of premature birth, and those studies have reported conflicting results. Thus, we cannot discount the possibility that physical activity may play a different role in the risk of extremely preterm birth than it does in the less severe and more common preterm birth subtypes.^35,36^ Physical activity patterns change with the seasons. However, warmer months in Denmark, which in our study were associated with the highest risk of extremely preterm birth, are likely characterized by greater physical activity owing to increases in leisure activities and commuting by bicycle. Modifiers associated with psychological stressors and inflammation may play a substantial role in the orchestration of parturition^37^ and may help to explain why seasonal variation in psychological stressors may be important for the occurrence of extremely preterm birth.^38^

The goal of the 2020 lockdown was to reduce transmission of SARS-CoV-2, and there is some support for an association between COVID-19 infection during pregnancy and higher rates of preterm birth.^39,40^ It is now well recognized that lockdowns around the world were also associated with reductions in the transmission of other infectious disease pathogens, such as influenza virus.^41^ However, the evidence in support of infections as risk factors for preterm birth is not robust. An association with preterm births has primarily been observed for genitourinary infections, such as chlamydia and chorioamnionitis, and it is not apparent whether these infections would vary with season to the same extent as those produced by respiratory pathogens.^42,43^

The prevalence of preterm birth found in our study was lower than that of many other countries. Global preterm birth prevalence has been reported to be 11.1% compared with 5.0% in some northern European countries.^44^ The preterm birth rate in the Danish National Birth Cohort was reported as 3.9%,^30^ which is consistent with the 3.4% preterm birth rate found in our study when taking into account that we did not include pregnancies resulting in multiple births, possible differences in the denominators used, and the ways in which pregnancies ending before 21 completed weeks of gestation were handled.

### Strengths and Limitations

This study has strengths. Its main strength was the use of a fetuses-at-risk approach for analysis of nationwide data. This approach contrasts with the time-series method, in which births are used as denominators, that has been used in many previous studies. Births have substantial seasonality, and if factors associated with the risk of preterm birth vary by season, confounding can occur.^45^ Weinberg et al^46^ assessed Norwegian data using a time-series analysis and observed substantial seasonality among preterm births, with peaks in winter and summer. More modest seasonality was found using a fetuses-at-risk approach that was restricted to pregnancies with gestational age assessed primarily through ultrasonography and adjusted for sociodemographic covariates and maternal smoking status.^46^

The study also has limitations. First, a reduction in extremely preterm births might be associated with a concomitant increase in stillbirths during the same period. We took this possibility into account by including information on stillbirths in the cohort and using a competing-risks approach.

Second, season of pregnancy onset vs season during gestation can be difficult to distinguish in preterm birth studies because of collinearity issues. However, if the COVID-19 lockdown and seasonality share similar risk factors, the sudden onset and relatively short period of the lockdown suggest an association with season of gestation rather than season of pregnancy onset.

Third, our findings of attenuated associations between seasonality and more moderately preterm births do not necessarily suggest different etiologic characteristics for these preterm phenotypes. A risk factor that has an association with gestational age in general might produce results that are consistent with our findings of attenuation. This phenomenon can occur when the distribution of gestational ages is shifted slightly to the left (ie, toward earlier preterm birth in a plot of the distribution of gestational age at birth) by an exposure. When comparing the distributions of exposed and unexposed populations, a relative association in the leftmost tail of the distribution, where extremely preterm birth is located, may be observed. This association would then become more statistically significant when categorizing gestational age. We also cannot discount the possibility of the presence of a risk factor that is specific to extremely preterm birth, which would act only on the leftmost tail of the gestational age distribution. However, the clinical and public health impact would be the same, suggesting the potential identification of a preventable risk factor for extremely preterm birth. The benefits of identifying preventable risk factors are substantial for both maternal and offspring health.^47^ Few studies have distinguished between subtypes of preterm birth and may thus have missed important risk factors associated with gestational age in general or with extremely preterm births only.

## Conclusions

This cohort study found that seasonality was associated with 2.8% of all extremely preterm births, which was not observed among preterm births occurring closer to term. Given that substantial morbidity and mortality are associated with extremely preterm birth, further research to identify mechanisms and specific preventable risk factors associated with the seasonality of this adverse perinatal outcome is warranted.

## Notes

Supplement. **eTable 1.** *ICD-10* Codes Used From the National Hospital Register **eTable 2.** Sensitivity Analyses of the Association Between Season During Gestation and Extremely Preterm Birth
