# Supplementary material for: Assessment of Seasonality and Extremely Preterm Birth in Denmark
Source: JAMA Netw Open. 2022 Feb 3;5(2):e2145800. doi: 10.1001/jamanetworkopen.2021.45800 (PMC8814911; doi:10.1001/jamanetworkopen.2021.45800)
Supplement: Supplement. — eTable 1. ICD-10 Codes Used From the National Hospital Register eTable 2. Sensitivity Analyses of the Association Between Season During Gestation and Extremely Preterm Birth [file jamanetwopen-e2145800-s001.pdf]

Supplementary Online Content

Hviid A, Laksafoss A, Hedley P, et al. Assessment of seasonality and extremely preterm birth in Denmark. *JAMA Netw Open*. 2022;5(2):e2145800. doi:10.1001/jamanetworkopen.2021.45800

eTable 1. *ICD-10* Codes Used From the National Hospital Register

eTable 2. Sensitivity Analyses of the Association Between Season During Gestation and Extremely Preterm Birth

This supplementary material has been provided by the authors to give readers additional information about their work.

© 2022 Hviid A et al. *JAMA Network Open*.

eTable 1. *ICD-10* Codes Used From the National Hospital Register

Variables	ICD-10 Codes
Abortive Outcomes	
Other Abortive Outcomes	O01, O02
Spontaneous Abortions	O03
Induced Abortions	O04, O05, O06, O07, O08
Pre-eclampsia[1]	O14, O15.0

(1) Klemmensen AK, Olsen SF, Osterdal ML, Tabor A. Validity of preeclampsia-related diagnoses recorded in a national hospital registry and in a postpartum interview of the women. Am J Epidemiol. 2007 Jul 15;166(2):117-24. doi: 10.1093/aje/kwm139. Epub 2007 Jun 7.

© 2022 Hviid A et al. *JAMA Network Open*.

eTable 2. Sensitivity Analyses of the Association Between Season During Gestation and Extremely Preterm Birth

	Extremely Pretem Births	Pregnancies under study	1,000,000 Fetal Weeks	Spring v Winter	Summer v Winter	Autumn v Winter
Complete case analysis with adjustment for BMI and smoking status*	1140	689,680	4.12	1.03 (0.87-1.22)	1.09 (0.92-1.29)	1.22 (1.03-1.44)
Considering only spontaneous live births	811	1,136,143	6.78	1.20 (0.98-1.47)	1.30 (1.06-1.59)	1.35 (1.11-1.66)
Adjusting for interpartum length**	1985	1,123,956	6.71	1.09 (0.96-1.24)	1.14 (1.00-1.29)	1.24 (1.09-1.41)
Excluding pregnancies with interpartum length <18 months	1812	1,083,878	6.47	1.08 (0.99-1.17)	1.05 (0.96-1.14)	1.12 (1.03-1.22)
Excluding pregnancies with pre-eclampsia	1764	1,103,600	6.58	1.05 (0.96-1.15)	1.07 (0.98-1.17)	1.14 (1.04-1.25)
Adjusting for pre-eclampsia status as a time varying variable	2009	1,136,143	6.78	1.12 (0.99-1.27)	1.15 (1.01-1.31)	1.24 (1.09-1.41)

* BMI in 6 groups (<18.5, 18.5-25, 25-30, 30-35, 35-40, 40-45, >45) and smoking in 3 groups (non-smoker, stopped smoking during pregnancy, smoker)
** Interpartum length in 4 groups (First pregnancy, <18mo, 18mo-4yrs, >4yrs)

© 2022 Hviid A et al. *JAMA Network Open*.
